# Multifocal Adult Rhabdomyoma of the Head and Neck Manifestation in 7 Locations and Review of the Literature

**DOI:** 10.1155/2013/758416

**Published:** 2013-06-13

**Authors:** Lorraine A. de Trey, Stephan Schmid, Gerhard F. Huber

**Affiliations:** Department of Otolaryngology, Head and Neck Surgery, University Hospital Zurich, Frauenklinikstraße 24, 8091 Zurich, Switzerland

## Abstract

*Background*. Adult rhabdomyoma is a rare benign tumour with the differentiation of striated muscle tissue, which mainly occurs in the head and neck region. Twenty-six cases of multifocal adult rhabdomyoma are documented in the literature. *Method*. We report a 55-year-old male with simultaneous diagnosis of 7 adult rhabdomyomas and review the literature of multifocal adult rhabdomyoma. *Result*. Review of the literature revealed 26 cases of multifocal adult rhabdomyoma, of which only 7 presented with more than 2 lesions. Mean age at diagnosis was 65 years with a male to female ratio of 5.5 : 1. Common localizations were the parapharyngeal space (36%), larynx (15%), submandibular (14%), paratracheal region (12%), tongue (11%), and floor of mouth (9%). Besides the known radiological features of adult rhabdomyoma, our case showed FDG-uptake in (18) F-FDG PET/CT. *Conclusion*. This is the first case of multifocal adult rhabdomyoma published, with as many as 7 simultaneous adult rhabdomyomas of the head and neck.

## 1. Introduction

Rhabdomyoma, named by Zenker [1] in 1864, is an exceedingly rare benign tumor that exhibits mature skeletal muscle differentiation. Although in general benign soft tissue neoplasms outnumber their malignant counterpart, this is not true for rhabdomyomas, which are considerably less common than rhabdomyosarcomas and account for no more than 2% of all striated muscle tumors. Topographically a distinction is made between the more common cardiac and the extracardiac localizations. Cardiac rhabdomyomas are rare tumors that occur chiefly in the heart of infants and small children [2]. They are considered to be hamartomatous lesions and are frequently associated with tuberous sclerosis [3]. According to Weiss and Goldblum extracardiac rhabdomyomas can be divided into adult, fetal, and genital types, the adult type being the most common [2]. Adult rhabdomyoma (ARM) predominantly occurs in individuals over 40 with a male to female ratio of 3 : 1 to 5 : 1 depending on the literature [2, 4, 5]. There is a predilection for the head and neck region. ARM mostly appears as a solitary lesion but may be multicentric in about 15% [6]. Fetal rhabdomyoma is even less common than ARM. It also mainly occurs in male patients in the head and neck but is often present at birth. Moreover, it differs from the adult type in its areas of predilection and histology. Two different subtypes are known, the myxoid and intermediate types. The genital rhabdomyoma is a rare tumor found in the vagina and vulva of middle-aged females. As a related lesion, Weiss further mentions the rhabdomyomatous mesenchymal hamartoma, a striated muscle proliferation that occurs mainly in the periorbital and perioral region of infants and young children [2].

In this study we present a case of multifocal ARM with simultaneous diagnosis of 7 lesions. To our knowledge this is the first patient reported to have ARM in more than 3 locations. Moreover, we review the literature on multifocal ARM.

## 2. Case Presentation

**Figure 1:**
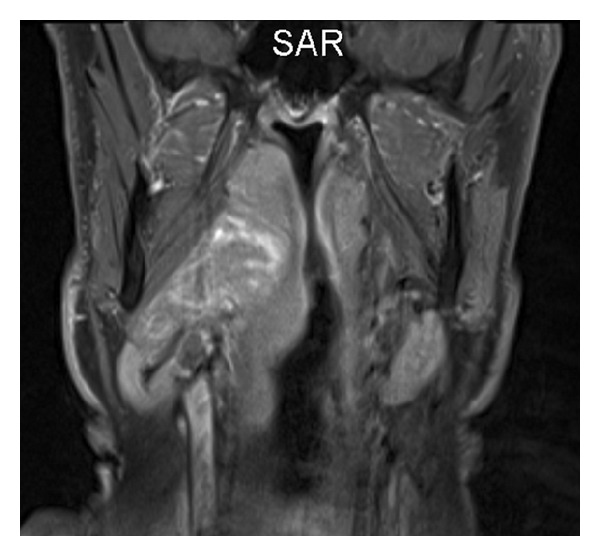
MRI ARM parapharyngeal space with submandibular extension.

**Figure 2:**
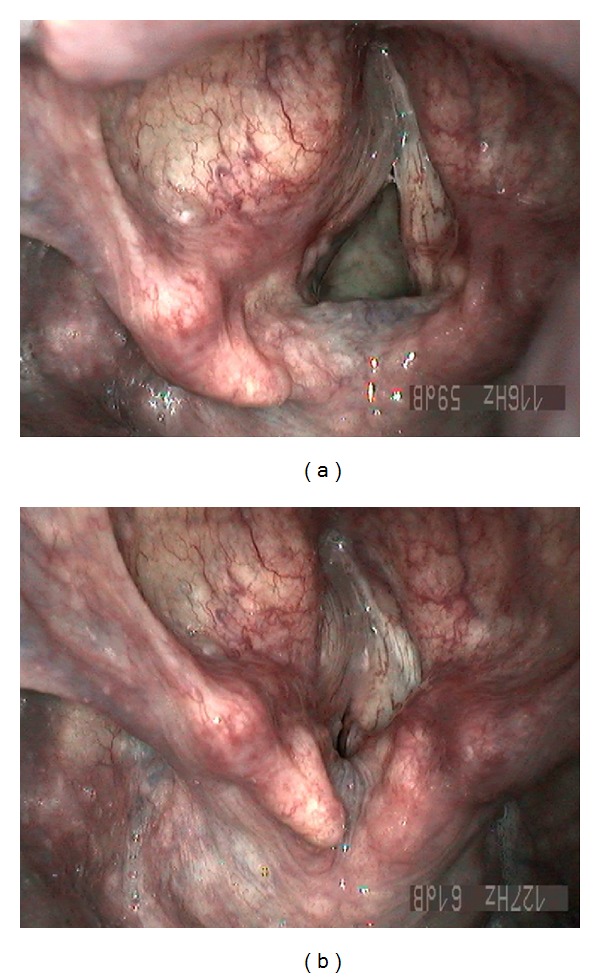
Laryngoscopy (inspiration and phonation) with left paraglottic ARM.

A 55-year-old male presented with a 3-month-history of hoarseness and slight dysphagia. His past medical history was significant for tonsillectomy and spontaneous pneumothorax. Physical examination showed an impressive asymmetry of the soft palate due to a right parapharyngeal mass. Moreover, there was a soft mobile submandibular mass palpable on the right. Magnetic resonance imaging (MRI) was performed which showed a large, homogenous, well-circumscribed parapharyngeal tumor on the right with a maximal diameter of 8.5 cm, extending inferiorly to the submandibular gland. A second tumor of smaller size was seen in the opposite parapharyngeal space. Both tumors had a similar aspect slightly hyperintense to muscle on native T1 and T2 with a light homogenous contrast enhancement (Figure 1). A biopsy of the right parapharyngeal tumor was taken transorally, which revealed the diagnosis of rhabdomyoma. The patient then was referred to our facility (tertiary referral centre). Besides the two parapharyngeal tumors, laryngoscopy showed a paraglottic mass of almost 2 cm on the left, covered by intact mucosa, which displaced the vocal cord medially and was responsible for the hoarseness. Vocal fold mobility was normal (Figure 2). On contrast enhanced computed tomography (CT), this mass again was well demarcated, homogenous, slightly hyperdense to muscle, and diffusely hyperattenuating. Three further small masses, around 1 cm in size, were seen in the floor of the mouth, tongue base, and in the retropharyngeal space. Ultrasound of the neck revealed additional bilateral retrothyroidal masses, 2 cm in diameter each, which so far had not been seen on imaging. They were well circumscribed, round, homogenous, and hypoechogenic. Fine needle aspiration cytology of the latter two lesions showed both to be rhabdomyomas.

**Figure 3:**
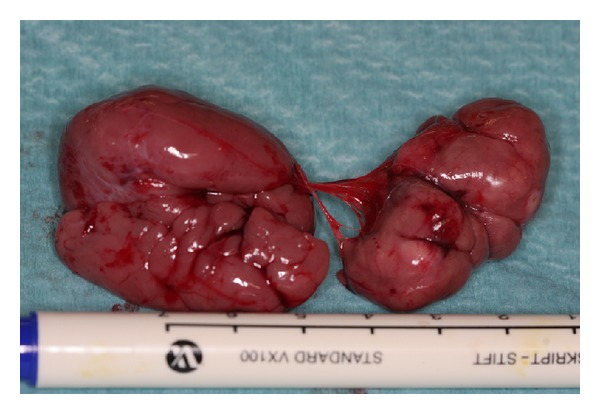
Right parapharyngeal ARM after excision.

Surgical removal of the parapharyngeal tumor on the right and the paraglottic mass on the left was performed, since they were responsible for the patient's symptoms. Both could be removed more easily than expected. The parapharyngeal mass was removed by a submandibular incision, from which the tumor could be released. Similarly, the paraglottic lesion could be removed endoscopically by an incision of the false vocal cords. Both tumors had a likewise appearance, brownish, soft, lobulated, with a smooth shiny surface (Figure 3). Histological examination confirmed ARM.

Bizon et al. [7] described that ARM in the (18) F-fluoro-2-deoxy-D-glucose ((18) F-FDG) PET/CT-scan has an elevated FDG-uptake. To confirm this information we performed (18) F-FDG PET/CT 3 months after the operation. It showed an elevated FDG-uptake in all previously diagnosed lesions with a maximal Standardized Uptake Value (SUVmax) of 2.9 including the 3 small lesions in the floor of the mouth, tongue base, and retropharyngeal space on which no histological examination was performed. Moreover, it demonstrated that the most cranial lobule of the parapharyngeal lesion on the right had not been removed. This lobule had been attached to the main tumor only by a fine strand of fiber. A transparotid approach would be necessary to remove this part of the lesion, which did not cause any symptoms to the patient postoperatively. Therefore, a further operation was not considered to be appropriate, neither was the removal of the other 5 ARMs.

## 3. Discussion and Review of the Literature

Adult rhabdomyomas are rare benign tumors with the differentiation of striated muscle tissue. They are considered to be true neoplasms unlike cardiac rhabdomyomas that are regarded as hamartomas [8]. Ninety percent of ARM are found in the head and neck region [4]. A reason for this predilection according to Weiss and Goldblum [2] is that the tumor arises from the branchial musculature of the third and fourth branchial arches. Adults with a median age between 55 and 60 are affected, predominantly males [2, 4, 5]. Patients most commonly present with a soft painless slow growing mass, sometimes with symptoms as globus sensation, hoarseness, or dysphagia. The clinical aspect of ARM is clearly benign but otherwise unspecific. Radiologically ARM presents as homogenous lesion that is isointense or slightly hyperintense to muscle on T1- as well as T2-weighted MRI and slightly hyperdense on CT. It enhances homogenously. Differential diagnosis depends on the location of the tumor and may include neurogenic or vascular tumors, oncocytoma, granular cell tumor, and rhabdomyomasarcoma [9]. Imaging findings usually suggest a benign lesion because of submucosal location and absence of invasion of surrounding soft tissues. However, lesions situated in the parapharyngeal space might also be mistaken for a malignant neoplasm of the minor salivary glands [10]. On CT scan ARM may mimic malignant tumors because they can appear to have indistinct borders blending into adjacent isodense muscles [11]. Another differential diagnosis on CT is malignant lymphoma due to its homogenous appearance.

The typical macroscopic description of the tumor is that of a soft, coarsely lobulated, tan-grey, well-circumscribed, or encapsulated lesion. The definitive diagnosis is mostly made histologically but several authors emphasize the utility of fine needle aspiration cytology in making the correct diagnosis. To confirm skeletal muscle differentiation immunohistochemical stains are necessary. Even though the histology of ARM is distinctive, it is often mistaken for a variety of other lesions, particularly granular cell tumor, as well as hibernoma, oncocytoma, and paraganglioma [2, 5].

The treatment of choice is surgery since ARMs are well circumscribed and easily removable by blunt dissection over a small incision. However, a recurrence rate from 16% to 42% has been described [5, 12]. No incidences of malignant transformation or spontaneous regression are known.

About 15% of the patients with ARM present with multifocal lesions [6].  Table 1 shows all cases of multifocal adult-type rhabdomyoma in the head and neck found by searching PubMed/Medline with the key words multifocal, multilocular, and multicentric rhabdomyoma in the English, German, French, and Spanish literature. Since 1948, including our patient, there have been 26 cases published. Excluded from this review were the reports by Albrechtsen et al. [34], Blaauwgeers et al. [35], Gibas and Miettinen [8], and Zhang et al. [36]. The former three have been mentioned in many past reviews; however, it has to presumed that the cases described were of multilobulated nature rather than multifocal. Of the 26 patients 7 had lesions in more than 2 locations, with a maximum of 7 ARMs in our patient. Mean age at diagnosis was 65 years (median 65) with a male to female ratio of 5.5 : 1. Often patients presented with a solitary mass and clinical or radiological examination then revealed further lesions. In few cases the second site became evident only years after the primary diagnosis. Common localizations were the parapharyngeal space (36%), larynx (15%), submandibular (14%), paratracheal region adjacent to the thyroid gland (12%), tongue (11%), and floor of the mouth (9%). All lesions, except in our case, were treated by surgical excision. Seven authors reported recurrence; therefore, the recurrence rate in this series is at least 27%. A reason for high recurrence rates could be that ARMs are multilobulated. The postoperative persistence of tumor lobules, which remain unnoticed perioperatively, because they were attached to the main lesions only by small strands of fibrous tissue, can present as recurrence in the years to come.

## 4. Conclusion

ARM is a very rare tumor with predilection for specific areas of the male head and neck. It has typical radiological as well as macroscopic characteristics and it shows elevated FDG-uptake in (18) F-FDG PET/CT. Definitive diagnosis is made by fine needle aspiration cytology or final histology and requires immunohistochemical stains. It is important that correct identification of the ARM is made in order to avoid an unnecessarily aggressive resection, yet providing potentially curative therapy. Even though not much literature exists we only recommend surgery of lesions that are symptomatic or cosmetically disturbing. Removal is often possible by a small incision, since ARM is well circumscribed and not adherent to adjacent structures. The occurrence of multiple lesions (multifocal and multilobulated) has to be considered, since most likely postoperative persistence of tumor lobules is the reason for the high recurrence rates documented in the literature. To our knowledge this is the only case published with as many as 7 simultaneous ARMs.

## Figures and Tables

**Table 1:** Reported cases of multifocal ARM of the head and neck.

Author, year	Age	Sex	Nr	Localization
Beyer and Blair, 1948 [13]	52	M	2	L floor of mouth L hypopharynx
Goldman, 1963 [14]	82	M	2	L sternohyoid muscle L true vocal cord
Assor and Thomas, 1969 [15]	59	M	2	L submandibular region R parapharyngeal space
Weitzel and Myers, 1976 [16]	56	M	3	L parapharyngeal space (2) R parapharyngeal space
Scrivner and Meyer, 1980 [17]	72	M	3	R base of tongue, L vallecula R parapharyngeal space
Neville and McConnel, 1981 [18]	58	M	2	R floor of mouth L supraglottis
Gardner and Corio, 1983 [19]	60	M	2	L submandibular region L endolarynx (posterior wall of ventricle)
Schlosnagle et al., 1983 [20]	65	F	3	L + R submandibular region base of tongue
Golz, 1988 [21]	81	M	2	R paratracheal region retrolaryngeal region
Bertholf et al., 1988 [22]	65	M	2	L floor of mouth L neck
Walker and Laszewski, 1990 [23]	76	M	3	Tongue, R neck L parapharyngeal space
Kapadia et al., 1993 [5]	59	M	2	larynx parapharyngeal space
Shemen et al., 1992 [24]	53	M	4	R parapharyngeal space, L floor of mouth R paratracheal region (retrothyroidal), L larynx
Shemen et al., 1992 [24]	75	M	2	R floor of mouth R parapharyngeal space
Fortson et al., 1993 [25]	71	M	2	R parapharyngeal space R submandibular region
Zbaren et al., 1995 [26]	64	M	3	R submandibular region L + R aryepiglottic fold
Vermeersch et al., 2000 [27]	66	M	2	L + R parapharyngeal space
Welzel et al., 2001 [28]	77	F	2	R parapharyngeal space R paratracheal region
Padilla Parrado et al., 2005 [29]	69	F	2	L parapharyngeal space anterior mediastinum
Liess et al., 2005 [6]	69	M	2	R submandibular region R epiglottis
Delides et al., 2005 [30]	59	M	2	R tongue R paratracheal region (retrothyroidal)
Koutsimpelas et al., 2008 [31]	72	F	2	L aryepiglottic fold R proximal Oesophagus (retrothyroidal)
De Medts et al., 2007 [32]	65	M	3	R base of the tongue, R floor of mouth R submandibular region,
Grosheva et al., 2008 [33]	45	M	2	Retropharyngeal space Left parapharyngeal space
Bizon et al., 2008 [7]	65	M	3	R parapharyngeal space, L base of tongue R submandibular region
Present case, 2013	55	M	7	R and L parapharyngeal space, R retropharyngeal space R and L paratracheal region (retrothyroidal) R floor of mouth, L base of tongue

Nr: number of tumors; M: male; F: female; R: right; L: left.
